# The Precision-Guided Use of PI3K Pathway Inhibitors for the Treatment of Solid Malignancies

**DOI:** 10.3390/biomedicines13061319

**Published:** 2025-05-28

**Authors:** Alexa E. Schmitz, Shirsa Udgata, Katherine A. Johnson, Dustin A. Deming

**Affiliations:** 1 McArdle Laboratory for Cancer Research, Department of Oncology, University of Wisconsin, Madison, WI 53705, USA; 2 University of Wisconsin Carbone Cancer Center, Madison, WI 53705, USA; 3 Division of Hematology, Medical Oncology and Palliative Care, Department of Medicine, University of Wisconsin School of Medicine and Public Health, University of Wisconsin, Madison, WI 53705, USA

**Keywords:** PI3K, AKT, MTOR, targeted therapy, colorectal cancer

## Abstract

Phosphatidylinositol-3-kinase (PI3K)/AKT/mammalian target of rapamycin (MTOR) pathway hyperactivation is seen in a multitude of malignancies. Due to the importance of this pathway in numerous critical cellular functions, preclinical and clinical investigations have aimed to target this pathway as an anti-cancer therapeutic strategy. This has led to the development of PI3K, AKT, and MTOR inhibitors for use in cancer patients, leading to multiple FDA approvals over the past decade. In this review, we outline therapeutic targets in PI3K/AKT/MTOR signaling in solid tumors, the current state of using inhibitors of this pathway to treat patients whose cancers possess activating mutations in *PIK3CA*, *AKT1/2*, or *MTOR*, and exciting new inhibitors that are entering clinical trials.

## 1. Introduction

The phosphatidylinositol-3-kinase (PI3K) pathway is frequently mutated in multiple cancers and is a key regulator of important functions such as cell proliferation, cell differentiation, cell survival/apoptosis, and metabolism [1,2,3]. PI3K is part of the lipid kinase family located at the plasma membrane of the cell and is divided into three different classes: class 1, class 2, and class 3 [3]. Class 1 PI3K has two subclasses, denoted as class 1A and class 1B, differentiated by which regulatory isoforms they have [2]. Class 1A has the isoform p85, while class 1B has p101 or p87 [2]. Additionally, class 1A is activated by receptor tyrosine kinases and G-protein-coupled receptors, while class 1B is only activated by G-protein-coupled receptors [4]. Currently, class 1A is the only PI3K subclass associated with tumorigenesis, and thus it will be the focus of this discussion [3].

**Figure 1:**
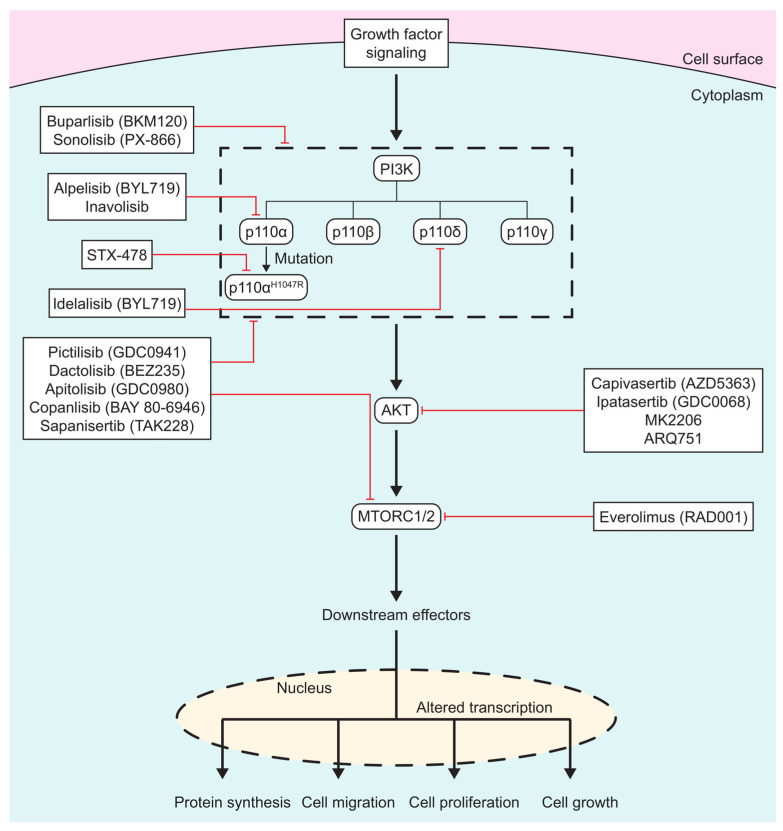
Scheme of PI3K/AKT/MTOR inhibitors: PI3K/AKT/MTOR inhibitors listed in boxes, with red lines signifying which protein(s) drugs inhibit. Specific isoforms of PI3K are outlined in the dashed box.

In the past 40 years, numerous PI3K/AKT/MTOR inhibitors have been developed, (Figure 1), with some leading to FDA approvals. There are three different classes of PI3K inhibitors: isoform-specific, pan-PI3K, and dual PI3K/MTOR inhibitors. Recently, there has been a shift in the development of PI3K inhibitors to targeting specific mutations in PI3K in order to overcome the side effects seen with the more generalized inhibitors. AKT inhibitors alter ATP binding to the activation loop of AKT. MTOR inhibitors are usually ATP analogs that prevent the binding of ATP on mTOR, thus shutting down its function.

## 2. PI3K/AKT/MTOR Pathway Overview

Class 1A PI3K has three isoforms—PI3Kα, PI3Kβ, and PI3Kδ—while class 1B has one isoform: PI3Kγ [4]. Class 1A isoforms are heterodimer proteins that have a catalytic subunit called p110 and a regulatory subunit called p85 [4]. The regulatory subunit, p85, is 85 kDa and has two SH2 domains bound to it that have been shown to interact with a multitude of different proteins [5]. p110 comprises an N-terminal adaptor-binding domain, a Ras-binding domain, a C2 domain, a helical domain, and a catalytic kinase domain [5]. Research indicates that the helical domain is important for regulating PI3K activation by inhibiting p85, and that mutations can lead to the loss of this inhibition [6]. The catalytic kinase domain of PI3K activates PIP3 by phosphorylating lipids on the cell membrane, leading to downstream activation [7]. Catalytic kinase domain mutations lead to the hyperactivation of the PI3K pathway [7]. There are three hotspot mutations found in these two domains: E545K and E542K, which are both located in the helical domain, and H1047R, in the catalytic kinase domain [2]. These mutations comprise up to 80% of *PIK3CA* mutations that are found in tumorigenesis [8].

When growth factors bind receptor kinases, PI3K becomes activated, leading to the formation of phosphatidylinositol (3,4,5)-triphosphate (PIP_3_) from phosphatidylinositol (3,4)-biphosphate (PIP_2_) [9]. PIP_3_ binds to 3-phosphoinositide-dependent kinase 1 (PDK1), which activates protein kinase B (AKT) [9]. AKT is a kinase that is part of the AGC kinase family and activates proteins important for protein synthesis, the progression of cell cycle, and p53 degradation [4,9]. AKT is a serine/threonine kinase that is vital for cell survival, cell cycle progression, p53 degradation, protein synthesis, and glucose uptake [9] AKT has three isoforms, which are denoted as AKT1, AKT2, and AKT3 and have three domains: a central kinase domain, an N-terminal fragment with a pleckstrin homology (PH), and a C-terminus fragment with a hydrophobic motif [10]. AKT1 has been shown to play an important role in cell proliferation and cell survival, AKT2 in cell metabolism and in regulating the cytoskeleton dynamics of the cell, and AKT3 in helping AKT1 with its involvement in mediating cell growth [10]. AKT activates mammalian target of rapamycin complex 1 (MTORC1), specifically the proline-rich AKT substrate of 40 kDa (PRAS40) subunit [9]. 

Once AKT is activated, it can control over 100 proteins and mediate multiple different pathways in the cell [10]. Some examples of proteins or pathways AKT is involved in are MTORC1, MDM2, NF-kB signaling, ERK signaling, and JNK signaling [9].

The mammalian target of rapamycin (MTOR) is a protein kinase that comprises two complexes, MTORC1 and MTORC2, and is important for protein and ribosomal biosynthesis, anabolism, and glucose metabolism [4,11]. MTORC1 is composed of five different subunits: regulatory associated protein of MTOR (Raptor), proline-rich AKT substrate 40 kDa (PRAS40), mammalian lethal with sec-13 (mLST8), MTOR, and Deptor [4,12]. The functions of these individual subunits are not well understood; however, it has been shown that PRAS40 acts as a negative regulator of MTORC1 [13]. MTORC1 is activated by amino acids and nutrients and can be phosphorylated by AKT to promote its upregulation [4,14]. MTORC1 has also been shown to be important for the regulation of protein synthesis and cell growth [14]. MTORC2 comprises the rapamycin-insensitive companion of MTOR (Rictor), mammalian stress-activated protein kinase interacting protein 1 (mSIN1), MTOR, and mLST8 [4]. Rictor and SIN1 bind to one another, hinting that their interaction is needed for the stability of MTORC2 [15]. mLST8 is important for the interaction between MTOR and Rictor; however, it is not known how it helps with this interaction [15]. Deptor is a negative regulator of MTORC2, such that the loss of this protein results in the upregulation of MTORC2 [15]. Interestingly, in myelomas, Deptor is upregulated when MTOR is upregulated, hinting that Deptor can be an activator of MTORC2 during tumorigenesis [14]. MTORC2 is important for cytoskeleton rearrangement, cell migration, cell proliferation, and the activation of AKT [4,15].

## 3. Targeting PI3K

### 3.1. PI3K Preclinical Data

There has been a multitude of preclinical evidence showing the effectiveness of PI3K inhibitors in cancers with the hyperactivation of the PI3K pathway. Medulloblastoma, a brain cancer found in children, is known to have an upregulation of the PI3K pathway, and when treated with pictilisib (GDC-0941), a PI3K class 1 selective inhibitor, cell migration and tumor growth is impaired [16]. Ovarian cancer cells, which commonly have a loss of expression of the PI3K pathway regulator PTEN, when treated with a PI3K inhibitor called D-116883, exhibit an increase in apoptosis and a decrease in cell growth [17]. In *PIK3CA*-mutant colorectal cancer lines, copanlisib, another class 1 selective inhibitor, has been shown to increase apoptosis, decrease cell growth, and decrease phosphorylated AKT expression in cells [18,19,20]. LY3023414, a dual PI3K/MTOR inhibitor, decreases cell proliferation in colorectal cancer cells as well as a reducing the growth of *PIK3CA*-mutant colon tumors in vivo [19]. 

### 3.2. PI3K Clinical Data

In 1957, the first PI3K inhibitor, wortmannin, was isolated and named after the fungus that it originated from, *Penicillium wortmannin* [21]. While this was a big step for studying PI3K inhibitors, this drug was not practical for clinical use due to its short half-life and severe side effects, like liver dysfunction, hyperglycemia, and lymphocytopenia [22]. LY294002, the next PI3K inhibitor to be developed, discovered by Eli Lilly in 1994, was more stable but was shown to be less potent than wortmannin [21]. Additionally, LY294002 was also not practical for clinical use due to its poor solubility and poor specificity for PI3K [16]. Wortmannin and LY294002 are considered “first-generation PI3K inhibitors” and were stepping stones for the development of future PI3K inhibitors.

The second-generation PI3K inhibitors are divided into three different classes: pan-PI3K inhibitors, isoform-specific PI3K inhibitors, and dual PI3K/MTOR inhibitors [23]. Pan-PI3K inhibitors target the four isoforms of class 1 PI3K enzymes, while isoform-specific PI3K inhibitors only target one specific isoform [24]. Dual PI3K/MTOR inhibitors differ from the other two drug classes by additionally targeting MTOR [24].

### 3.3. Pan-PI3K Inhibitors

Pan-PI3K inhibitors target PI3Kα, PI3Kβ, PI3Kδ, and PI3Kγ [24]. Examples of these types of inhibitors are copanlisib, buparlisib, pilaralisib, pictilisib, and sonolisib [24].

Copanlisib (BAY 80-6946) is a pan-PI3K inhibitor that primarily inhibits the PI3Kα and PI3Kδ isoforms [25]. Copanlisib has been involved in multiple clinical trials, and in 2022, patients with solid tumors that had a *PIK3CA* mutation were treated with copanlisib in a phase II clinical trial [25]. The study met its primary endpoint of an overall response rate (ORR) of 16%, showing that copanlisib was a promising inhibitor for patients with *PIK3CA*-mutant cancers [25]. The common side effects for patients treated with copanlisib were diarrhea, fatigue, decreased neutrophil and platelet counts, and hyperglycemia [26,27]. Copanlisib was FDA approved for patients with follicular lymphoma; however, in 2023, copanlisib lost FDA approval due to a lack of activity in further studies.

Pilaralisib (XL147) targets all class 1 PI3K isoforms and prevents the formation of PIP_3_ [28]. In a phase II clinical trial in patients with advanced endometrial carcinoma, pilaralisib had minimal anti-tumor activity but was tolerated well by patients [28]. Clinical trials have been performed in lymphoma, glioblastoma, endometrial, lung, breast, and ovarian cancers [NCT01943838, NCT01013324, NCT1240460, NCT00756847, NCT01082068].

Pictilisib (GDC-0941) is also an oral drug that inhibits class 1 PI3K isoforms and has been involved in multiple phase I/II clinical trials for solid tumors, primarily focusing on breast and lung cancer [4] [NCT01437566, NCT01493843, NCT00928330, NCT00974584]. A phase I clinical trial showed that pictilisib was well tolerated, with patients experiencing nausea, rash, and fatigue [29]. Pictilisib has been in phase II clinical trials in combination with cisplatin, paclitaxel, letrozole, and bevacizumab or trastuzumab. These trials have shown promise, with these combinations having anti-tumor effects in patients [30,31].

Sonolisib (PX-866) is a wortmannin analog that inhibits PI3Kα, PI3Kβ, and PI3Kδ isoforms [32]. In a phase II glioblastoma study, only 3% of patients met the primary endpoint of objective response and early progression [33]. In another phase II trial testing PX-866 on castration-resistant prostate cancer, 32% of patients showed progression-free survival (PFS) at 12 weeks, meeting the primary endpoint [34]. PX-866 has also been involved in phase I/II studies for colorectal, lung, and head and neck cancers; however, no results have been published on these studies at this time. Its common side effects include diarrhea, nausea, and lymphopenia [33].

### 3.4. Isoform-Specific PI3K Inhibitors

Isoform-specific PI3K inhibitors target only one isoform of PI3K. Examples of drugs that are in this class include alpelisib, idelalisib, and inavolisib [1].

Alpelisib (BYL719) is an oral drug that inhibits PI3Kα and is FDA approved for the treatment of *PIK3CA*-mutant metastatic breast cancer [1,35]. In a phase III study looking at the combination of alpelisib and fulvestrant, patients treated with the combination had a PFS of 11 months compared to 5.7 months with fulvestrant alone [36]. Due to this study, alpelisib was FDA approved in combination with fulvestrant for hormone receptor (HR)-positive, human epidermal growth factor receptor 2 (HER2)-negative, *PIK3CA*-mutant, advanced, or metastatic breast cancer [36]. The side effects associated with treatment with alpelisib include bladder pain, diarrhea, difficulty breathing, nausea, blurred vision, and bloating in the legs and arms [37]. 

Inavolisib (GDC-0077) inhibits the PI3Kα isoform and is given orally to patients [35]. In 2024, a phase III clinical trial, INAVO120, examined its use to treat *PIK3CA*-mutant, HR+, HER2−, locally advanced, and metastatic breast cancer [38]. When patients were treated with the combination of inavolisib, fulvestrant, and palbociclib, PFS was 15.0 months compared to 7.3 months with fulvestrant and palbociclib alone [38]. Over 58% of patients had a response to the combination, compared to 25% in the control group [38]. In October 2024, inavolisib was FDA approved in combination with fulvestrant and palbociclib in *PIK3CA*-mutant, HR+, HER2−, locally advanced, or metastatic breast cancer [39]. 

### 3.5. Dual PI3K/MTOR Inhibitors

An additional subclass of second-generation PI3K inhibitors is dual PI3K/MTOR inhibitors. Examples of these types of drugs are dactolisib, apitolisib, and samotolisib [3].

Dactolisib (BEZ235) is an imidazoquinoline that inhibits the ATP-binding domains of both PI3K and MTOR [3,40]. Dactolisib was involved in multiple phase I/II clinical trials, but the results of these trials were poor due to adverse effects, low tolerance, and minimal response [41]. The most common side effect of dactolisib is hair loss (alopecia) [42].

Apitolisib (GDC-0980) is a drug that is taken orally, inhibits both PI3K/MTOR, and has been in clinical trials for endometrial carcinoma, breast, prostate, and renal cancers [43]. However, in a phase II trial for patients with renal cell carcinoma, it had a lower PFS than patients treated with the MTOR inhibitor everolimus [44]. In a clinical trial for non-Hodgkin lymphoma and advanced solid tumor patients, 80% showed a decrease in tumor markers and many tolerated the drug well [45]. Its common side effects include rash, hyperglycemia, liver dysfunction, diarrhea, and fatigue [45].

Samotolisib (LY3023414) is an ATP-competitive inhibitor for both PI3K and MTOR. In a phase II clinical trial of patients with endometrial cancer with a PI3K pathway mutation, LY3023414 showed modest anti-tumor activity, with a PFS of 2.5 months [46]. Other phase II clinical trials have been or are currently being performed in osteosarcoma and castration-resistant prostate cancer [47,48]. Common side effects seen in patients receiving LY3023414 are anemia and hyperglycemia [48]. 

### 3.6. Resistance Mechanisms to PI3K Inhibitors

It is known that patients, when treated with PI3K inhibitors, have developed resistance to these inhibitors during the course of their treatment. This resistance can be caused by multiple different avenues, like the amplification of the mutated allele of PI3K, acquired mutations in PIK3CB, and other signaling pathways activating PI3K [49,50,51]. When studying the effects of PI3K inhibitors on breast cancer cells, it was determined using qPCR that the resistant cells had over 15 different copies of *PIK3CA*, compared to 2 in the parental line [50]. The effects of acquired *PIK3CB* mutations in response to *PIK3CA* inhibition was studied in breast cancer cells after treatment with pictilisib [51]. PTEN-null breast cancer cells were treated with pictilisib until they reached resistance and were sequenced to determine possible *PIK3CB* mutations [51]. It was determined that multiple clones of resistant PTEN-null breast cancer cells had a mutation in p110β D1067Y [51]. Lastly, one of the most common reasons for resistance to these inhibitors is other signaling pathways activating *PIK3CA*. One of the pathways that has shown the reactivation of *PIK3CA* is the PDK1-SGK1 signaling pathway [52]. In breast cancer cell lines that were resistant to alpelisib, the knockdown of PDK1 resensitized the cells to alpelisib [52]. This response was believed to be caused by PDK1 activating mTORC1 when PI3Kα inhibition occurred [52]. Another pathway that has been widely studied in its effect on PI3K inhibitor resistance is the RAS/MAPK pathway. In chronic lymphocytic leukemia, whole-exome sequencing was performed on patients after they had relapsed on PI3K inhibitors in clinical trials [53]. It was observed that 60% of patients had acquired mutations in *KRAS*, *BRAF*, or *MAP2K1* when they had reached resistance [53].

### 3.7. Future Direction of PI3K Inhibitors: Targeting Mutation-Specific PI3K

In the past 20–30 years, there has been a huge improvement in the formulation and application of PI3K inhibitors for patients. However, due to the severe side effects seen in patients due to these inhibitors affecting wild-type PI3K, there is still much improvement needed in the development of these inhibitors [24]. Currently, research is being conducted to make new inhibitors that are specific to a PI3K mutation, like H1047R or E545K. 

An example of one of these types of inhibitors is LOXO-783, which is specifically an inhibitor for the *PIK3CA^H1047R^* mutation. LOXO-783 is involved in a phase I clinical trial that is currently recruiting patients with a *PIK3CA^H1047R^* mutation in their cancer [54]. In this trial, LOXO-783 is being administered either as a monotherapy or in combination with one of the following: fulvestrant, imlunestrant, abemaciclib, paclitaxel, or an aromatase inhibitor [54]. 

Tersolisib (STX-478) is an allosteric second-generation inhibitor that is selective against *PIK3CA^H1047R^* mutation [55]. In vitro, tersolisib-treated adipocytes had a 50% decrease in the inhibition of glucose uptake compared to alpelisib [55]. In vivo, mice treated with tersolisib did not see a significant change in blood glucose levels compared to alpelisib, which saw a significant change [55]. Tersolisib was used to treat colon, lung, head and neck squamous cell carcinoma (HNSCC), and breast cancer xenografts, and showed that tumor growth inhibition was either similar to (breast and lung) or better than (colon and HNSCC) alpelisib [55]. A phase I/II clinical trial for patients with advanced solid tumors treated with tersolisib is underway, with patients with HR+ breast cancer being treated with tersolisib in combination with fulvestrant [NCT05768139]. Additionally, a clinical trial is being started to investigate the safety, tolerability, and breakdown of tersolisib in the body of healthy male patients [NCT06901336]. These clinical trials are still recruiting patients, and the trial results are eagerly awaited.

RLY-2608 is an allosteric, mutation-specific, PI3Kα inhibitor that has been shown to inhibit tumor progression and minimally impact insulin levels in vivo [56]. RLY-2608 affected cell lines that had either a kinase or a helical domain mutation due to a similar binding and disassociation with the p85 domain [56]. RLY-2608 was compared to alpelisib in PI3K-mutant breast cancer xenografts MCF7 (E545K), ST1056 (H1047R), and ST986 (E542K) [56]. In all the xenografts, RLY-2608 as a monotherapy resulted in a decrease in tumor volume [56]. In ST1056 and ST968, the combination of RLY-2608 with fulvestrant had an even bigger impact on the change in tumor volume compared to the control and RLY-2608 alone [56]. In a phase I ReDiscover trial, RLY-2608 had little impact on glucose homeostasis and showed anti-tumor activity in PI3Kα-mutant solid tumors [57]. 

With FDA approval for these new mutation-specific inhibitors, multiple clinical trials have been started on them, either as a monotherapy or in combination with other inhibitors [54,57] [NCT05768139]. One combination that seems to be widely used with PI3K inhibitors is its use alongside taxane-based chemotherapies like docetaxel and paclitaxel. In preclinical studies, the addition of capivasertib after docetaxel treatment has been shown to induce apoptosis and target surviving cells after docetaxel in prostate cancer cells [58]. Multiple clinical trials with either docetaxel or paclitaxel with capivasertib and alpelisib are currently ongoing [NCT05348577, NCT03997123, NCT02423603, NCT04216472, NCT02051751, NCT05660083]. Another combination therapy that is being looked at in a clinical trial is the combination of alpelisib with immunotherapy in metastatic breast and melanoma cancers [NCT06545682]. Additionally, with *PIK3CA*-mutant HER2+ being a common mutation profile in breast cancers, HER2+ inhibitors in combination with alpelisib are being investigated in multiple clinical trials [NCT04208178, NCT05230810, NCT02167854, NCT05063786].

## 4. Targeting AKT

### 4.1. AKT Preclinical Data

Preclinical data for AKT inhibitors has been promising. Borussertib, an AKT1 inhibitor, and a MEK inhibitor were used to treat both *KRAS*-mutant pancreatic and colorectal cancer cells, where the cells displayed anti-tumor activity [59]. In non-small-cell lung cancer (NSCLC), the use of the AKT inhibitor MK-2206 in combination with erlotinib, an EGFR inhibitor, showed synergy between the two inhibitors [60]. In vitro, twelve prostate cancer cell lines were injected into mice and then treated with either AZD5363 (a competitive ATP inhibitor for AKT), AZD8186 (PI3K beta and delta isoform inhibitor), or a combination of both [61]. After treatment, 10 of the 12 lines displayed inhibited tumor growth when treated with AZD563 [61]. When treated in combination with castration with either AZD81866 or AZD563 in vivo, long-lasting tumor regression was observed [61].

### 4.2. AKT Clinical Data

Currently, there are two classes of AKT inhibitors out on the market: competitive and allosteric AKT inhibitors [10]. Using AKT inhibitors as a monotherapy has been tested in clinical trials; however, their success has been limited [10]. The toxicity of these inhibitors seen in patients has been one of the ultimate drawbacks of these therapies, as well as the limited clinical response [10]. Currently, AKT inhibitors are being used in clinical trials in combination with chemotherapies and targeted treatments [10].

### 4.3. Competitive ATP Inhibitors

Competitive ATP inhibitors target all three isoforms of AKT and impact ATP binding to AKT [10]. Specifically, competitive ATP inhibitors bind to the ATP binding pocket located in the kinase domain of AKT [62]. Some examples of inhibitors in this class are capivasertib and ipatasertib [62].

Capivasertib (AZD5363) inhibits all three isoforms of AKT, specifically limiting the phosphorylation of AKT and PRAS40 in cells [63]. Multiple clinical trials have been conducted on capivasertib, including trials testing it as a monotherapy, in combination with other drugs, or in combination with chemotherapies [63] [NCT06613516, NCT06764186, NCT05593497, NCT04493853, NCT05348577, NCT03997123]. In a phase I clinical trial testing capivasertib as a monotherapy, patients who had breast, gynecological, or any *PIK3CA*/*AKT*-mutant solid malignancies were included [64]. At the conclusion of this study, it was determined that the best dosing for patients was 480 mg for 4 continuous days a week [64]. In 2023, a phase III clinical trial, CAPItello-291, showed that with the combination of capivasertib with fulvestrant in HER2- HR+ breast cancer, patients had a PFS of 7.2 months versus 3.6 months for the control group [65]. Capivasertib in combination with fulvestrant in HR+ HER2- breast cancer was FDA approved in 2023 [66].

Ipatasertib (GDC-0068) inhibits all isoforms of AKT by binding to the ATP binding pocket located in the kinase domain, stopping downstream signaling from occurring [67]. In 2022, an NCI match trial was published where patients who had *AKT1*^E17K^-mutant cancer were treated with ipatasertib; the study met its primary endpoint, with 22% of patients having a partial response (PR), 56% of them having stable disease (SD) and 9% having progressive disease (PD) [68]. Common side effects seen in patients were diarrhea, nausea, anorexia, increases in AST and creatinine levels, fatigue, hyperglycemia, and rash [68].

### 4.4. Allosteric AKT Inhibitors

Allosteric AKT inhibitors usually only have activity against AKT1 and AKT2 isoforms and not AKT3 [10]. Specifically, when AKT is in a closed conformation, the PH domain interacts with the kinase domain until AKT reaches the cell membrane where it is phosphorylated [69]. Once it has become phosphorylated, the PH domain and the kinase domain cannot interact anymore [13]. Allosteric inhibitors prevent this conformation from occurring, thus inhibiting AKT signaling [13]. Some examples of allosteric AKT inhibitors are MK-2206 and ARQ 751 [10].

MK-2206 has activity against AKT1 and AKT2 isoforms, with it working best in cells that have a PTEN mutation, AKT2 amplification, or RTK activation [10]. In a phase I trial where patients with a solid malignancy were treated with MK-2206, inhibitor treatment led to a decrease in AKT pathway signaling, though side effects were severe, leading researchers to look at testing MK-2206 in combination with other drugs [70]. The common side effects seen in this study were nausea, rash, fatigue, and diarrhea [70]. In a phase I clinical trial, cancer patients who had a HER2-overexpressing solid tumor were treated with MK-2206, paclitaxel, and trastuzumab [71]. After the study was completed, of the 16 patients involved in the study, 3 had CR, 7 had a PR, 1 had PD, 4 had SD, and 1 had non-CR/non-PD [71].

### 4.5. Future Directions for AKT Inhibitors

While capivasertib and ipatasertib have shown benefit in trials, the use of other AKT inhibitors as a monotherapy has had limited efficacy. Covalent allosteric AKT inhibitors (CAAIs) hold great potential [72]. CAAIs are like allosteric AKT inhibitors, with selectivity against AKT isoforms, but they also covalently modify the AKT activation loop, making an irreversible modification by changing out two cysteines [72]. Borussertib, which was discussed in the AKT Preclinical Data Subsection, is an example of a CCAI [72].

## 5. Targeting MTOR

### 5.1. MTOR Preclinical Data

Preclinical data for MTOR inhibitors has been promising over the past couple of decades. In renal cell carcinoma, a second-generation MTOR inhibitor, MLN0128, was used to treat realistic patient-derived tissue slice grafts and showed decreased levels of 4EBP1, c-Myc, and p-S6K1 [73]. MLN0128 treatment also showed a decrease in colorectal cancer organoid size and a reduction in size of *PIK3CA*-mutant colon tumors in vivo [20]. CC1-779, an MTOR inhibitor, was used to treat breast cancer cell lines and inhibited MTOR activity and decreased c-myc expression [74]. When compared to everolimus (a first-generation MTOR inhibitor) in breast cancer cells, NVP-BEZ235 (a dual MTOR/PI3K inhibitor) had higher antiproliferative activity [75]. NVP-BEZ235 has also been tested on multiple cancer cell lines (myeloma, human glioma, osteosarcoma, Ewing’s sarcoma, and rhabdomyosarcoma) and has been shown to decrease the phosphorylation of the PI3K pathway in all of them [76,77,78,79]. AZD8055 is an oral drug that has shown decreased cell proliferation in lung cancer cell lines (H383 and A549) and decreased phosphorylation in S6 and AKT in U87-MG, a PTEN-mutant glioblastoma cell line [80]. Nab-sirolimus (combination of sirolimus and everolimus), is a nanoparticle albumin-bound MTOR inhibitor which has shown promising preclinical data with increased MTOR suppression and decreased tumor growth [81].

### 5.2. MTOR Clinical Data

The first MTOR inhibitor was discovered in 1972 from bacteria called *Streptomyces hygroscopicus* and was called rapamycin [82]. Rapamycin makes a complex with the FK506-binding protein which then binds to MTORC1 and renders it inactive [82]. However, due to the drug’s poor water solubility and stability, it was not practical for clinical use [82]. To overcome this issue, a new inhibitor was created called temsirolimus, which converts to rapamycin when it enters the body [4]. Temsirolimus is given to a patient intravenously and has been FDA approved to treat patients with renal cell carcinomas [4,83]. Another drug that was created to overcome the issue of solubility and stability was everolimus. Everolimus has improved stability and solubility compared to rapamycin and is also FDA approved for patients with advanced renal cell carcinomas in combination with the tyrosine kinase inhibitor, lenvatinib [4,84]. Other drugs that are considered “first-generation” MTOR inhibitors are ridaforolimus, umirolimus, and zotarolimus [4].

The second generation of mTOR inhibitors comprises ATP analogs that are dual PI3K/MTOR inhibitors and selective MTORC1/2 inhibitors [11]. Selective MTORC1/2 inhibitors inhibit both MTORC1 and MTORC2 and are highly selective to these complexes [82]. Some examples of these drugs are TAK228, PKI-587, GDC-0980, and XL765 [11]. In an NCI MATCH phase II clinical trial, patients with a loss of function of tumor suppressor genes TSC1/2 when treated with sapanisertib (TAK228, a MTORC1/2 inhibitor) had a modest response to treatment, with 5 of the 34 patients having a PR and an estimated 6-month PFS of 28.7% [85].

Other clinical studies for MTOR inhibitors are looking at tuberous sclerosis complex (TSC) mutations. TSC is an autosomal genetic disorder that causes lesions in multiple different organs [86]. The two tumor suppressor genes that are commonly mutated are *TSC1* and *TSC2*, which leads to the hyperactivation of the MTOR pathway [86]. Nab-sirolimus has been FDA approved for advanced malignant perivascular epithelioid cell tumor (PEComa) after a registration phase II AMPECT study determined that 92% of patients had a response for more than 6 months [87].

### 5.3. Future Directions for MTOR Inhibitors

MTOR inhibitors have been promising for preclinical and clinical studies; however, due to negative feedback loops that occur with the incomplete inhibition of MTOR, drug resistance has become a problem [88]. The newest generation of MTOR inhibitors, denoted as the “third generation”, have been developed to overcome the resistance that has been seen in first- and second-generation drugs. Breast cancer cells were treated either with rapamycin or AZD8055 to obtain resistant colonies that had mutations either in the kinase domain or the FKBP12-rapamycin-binding domain [89]. Researchers determined that the rapamycin and TORKi (MTOR kinase inhibitors) binding sites were close to one another and that using an inhibitor that has a bivalent interaction could be useful to overcome this resistance [89]. An example of a drug in this category is Palomid 529 (P529). Recently, P529 has been shown to increase anti-tumor activity while also enhancing the efficacy of radiotherapy in prostate cancer cells [90]. There are currently no clinical trials of P529 for patients with cancer being conducted. Another example of a third-generation drug is RapaLink-1, which has been shown to be more potent than rapamycin and MLN0128 in glioblastoma cell lines [91]. RapaLink-1, which targets both rapamycin and INK-128 binding sites, is being used to overcome the issue of drug resistance [92]. Currently, there are no clinical trials for rapalink-1; however, it is expected that phase I clinical trials will start soon for the treatment of patients with renal cell carcinomas [93]. 

## 6. Conclusions

The PI3K pathway is instrumental in cell growth, cell proliferation, metabolism, and migration [1]. When this pathway becomes mutated, uncontrolled cell growth can occur, leading to tumorigenesis. Due to the importance of this pathway and the common oncogenic activation of this pathway, a multitude of inhibitors of this signaling cascade have been developed. There have been significant challenges in targeting this pathway clinically, but the successful FDA approvals of the PI3K inhibitors alpelisib and inavolisib and the AKT inhibitor capivasertib for PI3K-pathway-mutant breast cancer are a major step in the right direction (Table 1). Clinical trials, including arms of the MATCH trial, have also demonstrated significant potential for agents targeting solid tumors with PI3K pathway mutations. Further clinical trials are needed to expand the clinical experience of targeting cancers with activating mutations in the PI3K pathway. Additionally, future studies should explore combination regimens to enhance the sensitivity of these cancers to PI3K pathway inhibitors.

## Figures and Tables

**Table 1:** The PI3K/AKT/MTOR inhibitors summary table. For each inhibitor, its target, what cancer the drug is being studied in or what it is FDA approved for, the stage of development of the drug, and the side effects are given.

Inhibitor	Targets	Cancer Type	FDA Approval/Stage Development	Side Effects
Alpelisib (BYL-719)	PI3Kα	Breast Cancer [32]	FDA Approval in 2019 [32]	Nausea, blurred vision, bloating, diarrhea [34]
Capivasertib (AZD-5363)	AKT1/2/3	Breast Cancer [63]	FDA Approval in 2023 [63]	Diarrhea, nausea, increased glucose, vomiting [63]
Everolimus (RAD-001)	MTOR	Advanced Renal Cell Carcinomas [84]	FDA Approval in 2016 [84]	Hypertension, fatigue, diarrhea, hyponatremia [84]
Ipatasertib (GDC-0068)	AKT1/2/3	*AKT*^E17K^-Mutant Cancers [65]	Phase II [65]	Fatigue, hyperglycemia, rash, diarrhea [65]
Inavolisib (GDC-0077)	Pan-PI3K	Breast Cancer [36]	FDA Approval in 2024 [36]	Diarrhea, fatigue, nausea, decreased appetite, decreased neutrophils [36]
Pictilisib (GDC-0941)	Pan-PI3K	Triple-Negative Breast Cancer [27]	Phase II [27,28]	Nausea, rash, fatigue [26]
Sapanisertib (MLN0128, INK128, TAK-228)	MTOR	Cancers with TSC1/2 Mutations [85]	Phase II [85]	Fatigue, nausea, anemia, diarrhea [85]
Tersolisib (STX-478)	*PIK3CA*^H1047R^ mutation	Advanced Solid Tumors with *PIK3CA*^H1047R^ Mutation [48]	Phase I/II [48]	No data available
