# Correction: Schmitz et al. The Precision-Guided Use of PI3K Pathway Inhibitors for the Treatment of Solid Malignancies. *Biomedicines* 2025, *13*, 1319

**DOI:** 10.3390/biomedicines13112627

**Published:** 2025-10-27

**Authors:** Alexa E. Schmitz, Shirsa Udgata, Katherine A. Johnson, Dustin A. Deming

**Affiliations:** 1 McArdle Laboratory for Cancer Research, Department of Oncology, University of Wisconsin, Madison, WI 53705, USA; 2 University of Wisconsin Carbone Cancer Center, Madison, WI 53705, USA; 3 Division of Hematology, Medical Oncology and Palliative Care, Department of Medicine, University of Wisconsin School of Medicine and Public Health, University of Wisconsin, Madison, WI 53705, USA

**Correction**

The original article cited the following retracted reference: “18. Yan, J.; Yang, S.; Tian, H.; Zhang, Y.; Zhao, H. Copanlisib promotes growth inhibition and apoptosis by modulating the AKT/FoxO3a/PUMA axis in colorectal cancer. *Cell Death Dis*. **2020**, *11*, 943.”

Replacements have been applied for the following:

18. Destefanis, R.A.; Schmitz, A.E.; Steimle, A.K.; Payne, S.N.; Sha, G.C.; Olson, A.M.; Cornelio, A.; Lippert, A.E.L.; Kraus, S.G.; Johnson, K.A.; et al. BCL-2 Family Inhibition Enhances mTORC1/2 Inhibition in PIK3CA-Mutant Colorectal Cancer. *Mol. Cancer Ther.* **2025**, OF1–OF14. https://doi.org/10.1158/1535-7163.MCT-24-1096.
19. Udgata, S.; Stoecker, J.N.; Shen, X.; Pasch, C.A.; Deming, D.A. Co-existent PIK3CA and ARID1A mutations lead to enhanced sensitivity to PI3K inhibition through PUMA induction by FOXO3a and p53/p21. In *Proceedings of the American Association for Cancer Research Annual Meeting 2025, Part 1 (Regular Abstracts), Chicago, IL, USA, 25–30 April 2025*; AACR: Philadelphia, PA, USA, 2025; p. 371.

With this correction, the order of some references has been adjusted accordingly. The authors state that the scientific conclusions are unaffected. This correction was approved by the Academic Editor. The original publication [1] has also been updated.
